# Different Immunoregulation Roles of Activin A Compared With TGF-β

**DOI:** 10.3389/fimmu.2022.921366

**Published:** 2022-06-14

**Authors:** Fanglin Li, Yiru Long, Xiaolu Yu, Yongliang Tong, Likun Gong

**Affiliations:** ^1^State Key Laboratory of Drug Research, Shanghai Institute of Materia Medica, Chinese Academy of Sciences, Shanghai, China; ^2^University of Chinese Academy of Sciences, Beijing, China; ^3^Zhongshan Institute for Drug Discovery, Shanghai Institute of Materia Medica, Chinese Academy of Sciences, Zhongshan, China

**Keywords:** activin A, TGF-β, activin A signaling, macrophages, dendritic cells, B cells, T effector cells, regulatory T cells

## Abstract

Activin A, a critical member of the transforming growth factor-β (TGF-β) superfamily, is a pluripotent factor involved in allergies, autoimmune diseases, cancers and other diseases with immune disorder. Similar to its family member, TGF-β, activin A also transmits signals through SMAD2/SMAD3, however, they bind to distinct receptors. Recent studies have uncovered that activin A plays a pivotal role in both innate and adaptive immune systems. Here we mainly focus its effects on activation, differentiation, proliferation and function of cells which are indispensable in the immune system and meanwhile make some comparisons with those of TGF-β.

## Introduction

Activin A was initially defined as a factor which could induce the gonad to secrete follicle-stimulating hormone (FSH) (1). Encoded by *inhibin β A* (*INHBA)*, activin A is a homodimer of two inhibin β A subunits, and is referred to as the predominant member of activin branch of the transforming growth factor-β (TGF-β) superfamily (2). Theoretically, dimers composed of arbitrary two inhibin β subunits could exist, such as homodimer activin B and heterodimer activin AB (2), however, the vast majority of research has been carried out on activin A.

In the last few decades, activin A has been proven to be involved in a variety of biological processes apart from its original function, including hematopoiesis, tissue repair, angiogenesis and immune regulation (3, 4). In the aspect of immune regulation, activin A plays an important role in the development of diseases such as allergies, autoimmune diseases and cancers (5). However, how activin A modulates the immune system remains controversial and needs further exploration.

This review focuses on the effect of activin A signaling on the major components of the immune system. In view of the fact that it shares a classical downstream pathway with TGF-β, we choose to elaborate the difference between their functions throughout the paper.

## Activin A Signaling Pathway

All the members of TGF-β superfamily transmit signal *via* a serine/threonine kinase receptor system which involves seven type-I and five type-II receptors (6). The formation of receptor complex needs at least one type-I and one type-II receptor. Even though each TGF-β superfamily member favors a certain receptor complex, promiscuity or overlap of ligand-receptor interactions exists since any one of the 12 receptors binds to more than one ligand (6). The predominant receptors of activin A include activin receptor like kinase 4 (ALK4, type I) and activin receptor type IIB, activin receptor type IIA (ActRIIB, ActRIIA, type II) (2). The canonical downstream pathway of activin A is identical to that of TGF-β. Activin A first binds and promotes phosphorylation of type II receptors, and then recruits type I receptors to form phosphorylated heteropolymers. Activated receptor complex will phosphorylate mothers against decapentaplegic (SMAD) 2 and 3 at their carboxyl-terminal SSXS motif (6). Afterwards, SMAD4 is recruited to help form a transcriptional complex, which then translocates to the nucleus and affects transcription of genes Pax-6, FSH, p21 and follistatin (3, 7, 8). Apart from SMAD2/3, SMAD1/5/8 is also involved in the signaling of TGF-β superfamily members such as bone morphogenetic protein (BMP)s (9).

Some means can be used to intervene activin A signaling for the mechanism study. Activin A is strictly modulated by various molecules physiologically. Follistatin, a natural ligand of activin A, can bind to activin A with high affinity and is most commonly used to block the activity of activin A (10–13). Besides, a heterodimer comprised of inhibin α (INHA) and inhibin β (INHB) called inhibin, which also belongs to the activin family, is able to suppress activin A signaling by binding to activin A directly or competing for type II receptor (10). Antibodies designed for receptors are the major exogenous interventions. Notably, most of the TGF-β superfamily members bind to ActRIIB or ActRIIA while only a few ligands bind to ALK4 with high affinity, even excluding activin B and activin AB (14). In order to avoid nonspecific blockage as far as possible, it is better to use ALK4 inhibitor. For example, SB-431542 is the one that has been widely used in scientific research (11, 15–17).

**Figure 1:**
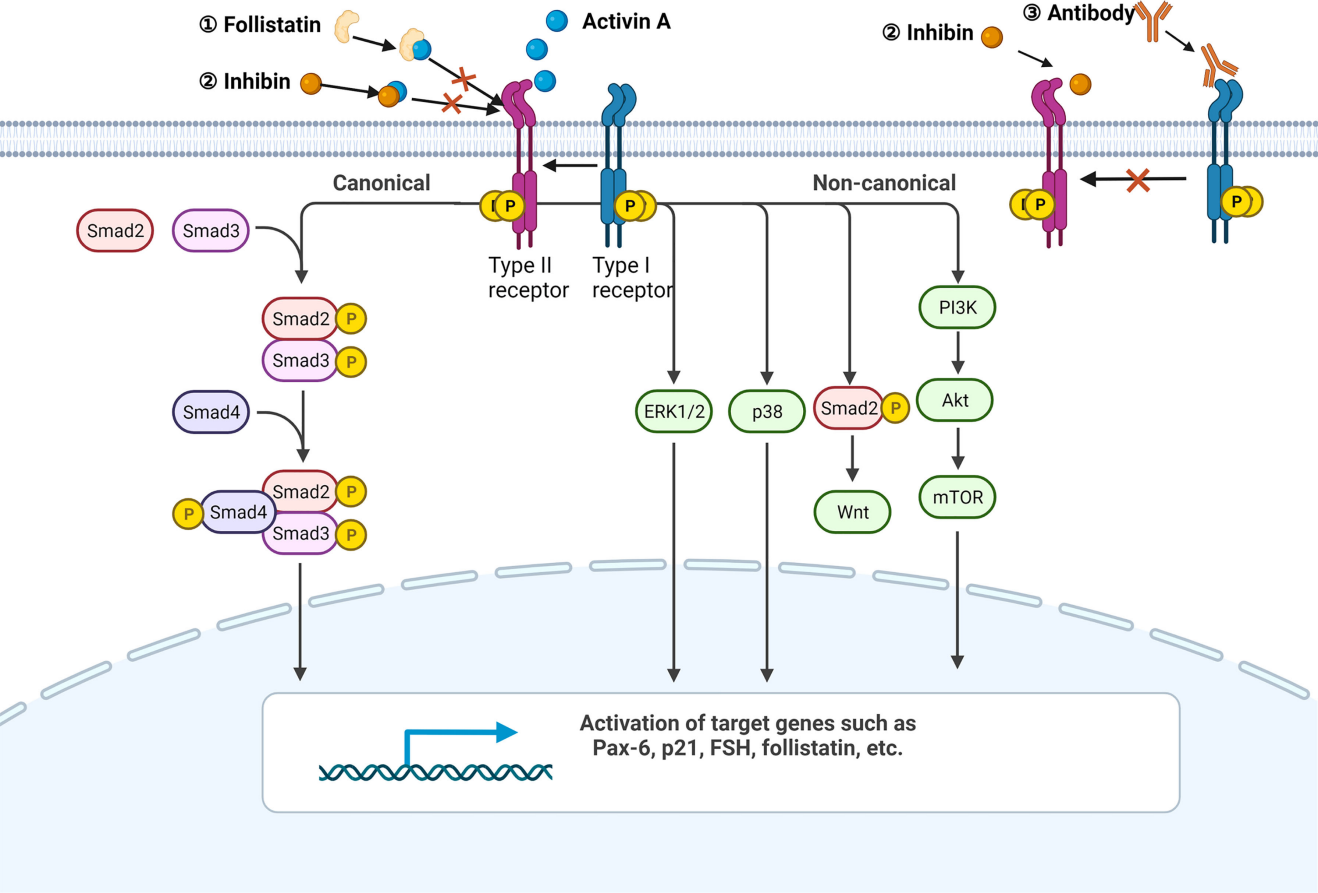
Activin A signaling pathway. A simplified illustration of the canonical and non-canonical signaling pathways of activin A. All the pathways are initiated by the formation of the activin A-heteropolymer receptor complex. The canonical pathway is Smad-dependent, while the non-canonical pathway can be mediated by ERK1/2, p38, Wnt, or PI3K. Endogenous ligand follistatin and activin family member inhibin can both bind to activin A directly to avoid the formation of activin A-heteropolymer receptors complex. Inhibin can also compete for the type II receptor. Antibodies targeting ALK4 are able to suppress downstream pathways as well. Created with BioRender.com.

In addition to the canonical receptor serine/threonine kinase-SMAD axis, activin A has also been shown to be involved in p38, ERK, PI3K, Wnt and other pathways (**Figure 1**) (18–21).

## Activin a Regulation of the Innate Immune System

In general, innate immune cells including macrophages, dendritic cells (DCs) and natural killer (NK) cells are the sources of activin A (12, 22–24). Here, we describe the relationship between activin A and these cells, and make some comparison with those of TGF-β.

### Monocytes/Macrophages

Although it has been demonstrated that tumor necrosis factor (TNF)-induced activation of RAW264.7, a macrophage cell line derived from BAB/14 mice, did not influence expression of activin A (22), an earlier study showed that *INHBA* could be up-regulated by LPS-induced human peripheral blood monocytes (23), suggesting that the activated monocytes/macrophages in specific conditions could be the source of activin A.

When macrophages are activated, secretion of pro-inflammatory cytokines including IL-1β and IL-6 will be increased, and cell surface markers like CD14 and CD80 will be up-regulated (25). It was reported that activin A could induce macrophages to release nitric oxide (NO), IL-1β, IL-6 and TNF, and up-regulate the expression of CD80, CD86 and CD14 on the cell surface as well (22, 26, 27). However, the above studies all used resting macrophages as research subjects, while in immune disorders, macrophages are usually activated (25). In the latter case, the results are contrary to previous ones. Activin A inhibited production of NO by LPS-activated mouse peritoneal macrophages and also down-regulated the expression of CD14 (28). Moreover, activin A could significantly down-regulate the expression of CD80/CD86 and inhibit secretion of cytokines including IL-1β, IL-6 and TNF-α by peripheral blood mononuclear cell (PBMC)-derived monocytes from acute Kawasaki disease patients (29).

For innate immunity, macrophages exert functions of phagocytosis and pinocytosis, while they play a role in presenting antigens to lymphocytes for adaptive immunity (25). Studies have shown that activin A can facilitate the pinocytic and phagocytic activities of both mouse peritoneal macrophages and RAW264.7 (26, 27). Change of the major histocompatibility complex (MHC) expression was uncertain in resting macrophages after being exposed to activin A (26, 27). Similarly, activin A plays a different role in LPS-activated mouse peritoneal macrophages by inhibiting phagocytosis and down-regulating expression of MHC-II (28). Lower percentage of MHC-II^+^ CD206^+^ cells in testicular leukocytes from Inha^+/-^ C57BL/6 mice with elevated activin A supports the above phenomenon (30). Besides, whether activated or not, activin A did not affect the proliferation of macrophages (26–28). Overall, these lines of evidence suggest that activin A could facilitate activation and phagocytosis of resting macrophages and exert the opposite function in the activated macrophages. In contrast, TGF-β tends to serve as an immunosuppressive cytokine which suppresses activation and antigen presentation function of monocytes/macrophages (31). It is likely that activin A boosts priming of a macrophage response, while maintains the homeostasis when macrophages are over activated.

Several researches focused on whether activin A, could skew macrophage toward M2 type. It was reported that activin A could up-regulate M2 signature arginase-1 expression on RAW264.7 (32). Furthermore, kidney of mice exposed to androgen was found to release higher levels of activin A, which was accompanied with the polarization of renal macrophages to M2 type (33). Finally, compared with wildtype mice, the proportion of MHC-II^-^CD206^+^ cells in testicular leukocytes from Inha^+/-^ C57BL/6 mice was also declined (30). All of these results indicated that activin A is related to M2-biased macrophages polarization. Although it was shown that TGF-β secreted by mesenchymal stem cells (MSCs) induced M2-like polarization of macrophages, defining TGF-β as a contribution factor for promoting M2 polarization currently still lacks evidence (34).

In addition, activin A has been found to act in human monocyte chemotaxis (35) and participate in crosstalk between macrophages and tumor cells (15). *INHBA* was up-regulated in melanoma-treated monocytes, and the exposure of activin A to human PBMC-derived monocytes and melanoma-treated monocytes, rather than wide type monocytes, could directly promote the transcription of protumoral cytokines such as *CCL20*, *TNF* and *VEGFA*, as well as immunosuppressive cytokines such as *IDO-1* and *PTGS2*. Conditioned media of melanoma-treated monocytes could inhibit T cell proliferation and advance the invasion of melanoma cells. Administration of SB-431542 abolished these phenomena, while direct exposure to activin A showed no observable alterations. Such results suggested that melanoma cells could interact with monocytes through activin A to indirectly affect the tumor microenvironment (TME). Besides, activin A was enriched in tumor associated macrophages (TAM) of melanoma patients and its expression level in TAM was significantly associated with a worse prognosis for melanoma patients (15).

### Dendritic Cells

Expression of both activin type I and type 2 receptors exists on the surface of dendritic cell and exogenous activin A can activate DC *via* SMAD2 or ERK1/2 signaling (36). After stimulation by CD40L/LPS, the mRNA levels of ALK4, ACTRIIA and ACTRIIB in human monocyte-derived DC (MoDC) changed with the extension of time, accompanied by increased secretion of activin A (12). While other members in the TGF-β superfamily, such as Nodal, TGF-β, BMP4, BMP7 and myostatin, remained unchanged during this process (12). These lines of evidence support that DCs are not only donors but also targets of activin A.

Blocking MoDC endogenous activin A after CD40L stimulation resulted in significant up-regulation of cytokines like IL-6 (12). As TGF-β is able to induce mouse antigen presenting cell (APC) to release BAFF (37), which can indirectly modulate immunoglobulin production of B cells, activin A could induce DCs and macrophages to secrete BAFF *via* ALK4/Smad3 pathway (36, 38). However, activin A was not found to impact on growth of DCs (38). Numerous studies have shown that TGF-β can inhibit maturation and antigen presentation function of DCs (39–42), while whether activin A plays a similar role to TGF-β is uncertain.

DCs require collaboration with other immune cells to function sophisticatedly. Although TGF-β is known to be an inhibitor of NK function (43), production of TGF-β by DCs was not found. It was confirmed that human NK cells expressed receptors of both activin A and TGF-β (44). Addition of activin A on the basis of IL-2 plus IL-12 stimulation to human NK cells gave rise to up-regulation of ALK4 together with down-regulation of TGF-BRII and TGF-BRI mRNA (44). Strong up-regulation of activin A and no change of TGF-β was observed in the NK-DC co-cultured system, and it was demonstrated that DC but not NK could contribute to production of activin A (11). Activin A elevation was blocked by neutralizing antibodies of various cytokines including granulocyte-macrophage colony stimulating factor (GM-CSF), TNF-α, interferon-γ (IFN-γ) and IL-1β, which implied that cytokines exposure was responsible for the up-regulation of activin A in the co-cultured system (11). Given that activin A could not be released by NK and activin A blockage boosted NK cell IFN-γ production prior to IL-12 plus Poly I:C stimulation in a co-cultured MoDC-NK system, activin A might be the mediator for MoDCs to regulate NK activity (44). Moreover, incubation of follistatin or SB-431542 in the co-cultured system significantly promoted secretion of IL-6, TNF-α and other DC-derived cytokines, while BMP inhibitors showed no effect (11). Additionally, activin A may serve as a regulator of DC maturation triggered by NK, since CD83 and CD86 were up-regulated following activin A inhibition in an NK-DC co-culture system (11).

Activin A also affected the effect of DCs on T cells. Different from TGF-β, which is known to exert immunosuppressive function by up-regulating regulatory T cells (Tregs) (45), activin A-stimulated DC mixed with T cells would not cause the up-regulation of CD25^+^Foxp3^+^ regulatory T cells (36). It was also shown that activin A could promote proliferation and inhibit apoptosis of T cells *via* BAFF and APRIL respectively (36). Whether these two factors are involved in differentiation and functions of T cells has not been reported. As tumor immunotherapy has become a hot spot in tumor research in recent years, the researchers fed activin A-treated DC back to B16/LLC tumor bearing mice to figure out whether activin A-treated DC could affect tumor progress *in vivo* (36). As a consequence, tumor progression was greatly inhibited and this function could be abolished by BAFF/APRIL knockdown (36). *In vitro*, activin A-treated DC also facilitated IFN-γ production from mouse splenic T cells (36). Considering that exposing DC to TGF-β-induced Foxp3^+^ Treg could weaken DC’s ability to stimulate naïve T cell immune responses (46), activin A induced-Foxp3^-^IL-10 producing Treg (Activin A-iTreg) was generated. Activin A-iTreg was able to inhibit DCs to prime Th2 response *in vivo*, presented by reduced release of IL-4 and IL-13 (47).

### Natural Killer Cells

NK cells play roles as both activin A donors and activin A target cells in certain context. *INHBA*, *ACVR2B*, *SMAD2/3/4* were all shown to be expressed in mouse peripheral blood NK cells (24). Stimulation of these cells with IL-2 *in vitro* could promote the release of activin A in a dose-dependent manner (24).

It was reported that exogenous activin A or TGF-β both could inhibit IFN-γ production in human NK cells (44). Apart from IFN-γ, addition of activin A to NK cells stimulated with IL-12 plus Poly I:C also led to the down-regulation of cytokines including IL-6, TNF-α, GM-CSF and IL-1β together with chemokines including MCP-1, IL-8 etc. (44). Moreover, IL-10 expression in NK cells was not altered after activin A administration, while IL-2 expression was up-regulated (24, 48).

Activin A was found to have no effect on the expression of perforin, granzyme A, granzyme B, IL-12RBI, and IL-12RBII in IL-2/IL-12 stimulated human NK cells. In contrast, TGF-β significantly suppressed the secretion of granzyme B, IL-12RBI, and IL-12RBII (44). It was also pointed out that the lytic function of NK was not affected by activin A (44). Instead, activin A was considered to be an inhibitor of NK cell proliferation, as well as cytotoxicity by inhibiting the release of LDH with a decreased killing rate of YAC-1 cells (13, 24, 44).

Rautela, J. et al. compared the effects of activin A and TGF-β on NK cells (13). Despite activin A might inhibit proliferation, cytokine secretion and cytotoxicity of NK cells, its function always tended to be inferior to TGF-β (13). However, treating TGF-βRII-deleted mice with melanoma with follistatin could further reduce melanoma lung metastasis, indicating that activin A functioned independently from TGF-β (13).

## Activin a Regulation of the Adaptive Immune System

The suppression effect of TGF-β for adaptive immunity has been widely reported. Whether activin A shows a similar function is the main topic in the following sections.

### B Lymphocytes

B cells also serve as donors and targets of activin A (49). Pretreatment of naïve B cells with activin A before LPS stimulation resulted in enhanced proliferation and immunoglobulins (Ig) production ability of B cells. However, co-treatment of activin A and LPS did not affect B cell proliferation and Ig production, which implied that only resting B cells responded to activin A (49, 50). The potential reason accounting for this might be the down-regulation of activin A receptor in LPS-activated B cells (49). Different from activin A, TGF-β suppresses proliferation, Ig production and survival of B cells (49, 51).

Activin A, like TGF-β, promoted IgA secretion by B cells, according to Lee, H. J. et al. (50). Notably, this effect could not be reversed by TGF-β antibody (50). Activin A was reported to be involved in Th2-type responses, which are characterized by IgE production. Even though IgE production by B cells could not directly be increased by activin A *in vitro*, activin A neutralization *in vivo* significantly decreased IgE production in mice immunized with OVA (49). It was possible that activin A indirectly induced IgE production by B cells with the help of IL-4 released from other immune cells such as macrophages (49).

Increased secretion of activin A by inflammatory monocytes could subsequently activate APC to release BAFF (38). Small-molecule drug-P_4_N was able to induce activin A production by monocytes, thereby promoting B cell proliferation and antibody production *via* activin A/BAFF pathway (52).

### T Effector Lymphocytes

TGF-β is known to be an immune suppressive factor for inhibiting proliferation, survival, cytokine secretion and differentiation of T cells (53). Ogawa, K. et al. showed that activin A was unable to make a difference in proliferation of CD4^+^ CD25^-^ T cells (32). However, activin A significantly reduced proliferation of human T cells and percentages of effector-producing CD4^+^ T cells, but greatly up-regulated IL-10^+^ CD4^+^ T cells (54). Blockade of TGF-β did not affect the function, demonstrating that activin A functions independently (54).

TGF-β can suppress the activity of CD8^+^ T cells (31), while the effects of activin A on CD8^+^ T cells are rarely explored. Similar to TGF-β blockade, knockdown (KD) of activin A induced CD8^+^ T cells priming during radiation therapy of 4T1 tumor-bearing mice, and combination of TGF-β blockade with acitivin A KD significantly augmented this effect (55, 56). Activin A could also inhibit the activity of CD8^+^ T cells in peripheral blood of acute Kawasaki disease patients (57).

Both activin A and TGF-β are involved in tumor immunology. In accordance with existing research and properties of TGF-β, activin A may also suppress tumor immune microenvironment. In contrast, Morianos, I. et al. provided evidence which might uncover activin A’s role as an anti-tumor factor (58). Treatment of OVA-expressing Lewis Lung Carcinoma mice with activin A attenuated tumor progression and heightened the ratio of tumor-infiltrating effectors to regulatory CD4^+^ T cells (58). The expression of immune checkpoints including programmed cell death protein 1(PD-1), lymphocyte-activation gene 3 (LAG-3), cytotoxic T-lymphocyte-associated protein 4 (CTLA-4) on tumor-infiltrating CD4^+^ T cells was reduced as well (58). Adoptive transferring of activin A-treated lung tumor-infiltrating CD4^+^ T cells into CD4 (-/-)-tumor-bearing mice could suppress tumor progression without CD4^+^ T cell exhaustion (58).. All of these results indicated that activin A might be a novel therapeutic molecule for lung cancer.

Multiple studies described activin A as a Th2 cytokine, as it mediates Th2-priming immune responses in allergic airway disease and some other diseases (4, 32, 59–61). When CD4^+^ T cells differentiated into Th cells, the release of activin A was increased (32). The mRNA level of *INHBA* was more abundant in Th2 rather than Th1 cells (32). Compared with activated Th1 cells, activated Th2 cells secreted higher levels of activin A (32).

Activin A is also associated with Th17 pathogenicity. Experimental autoimmune encephalomyelitis (EAE) and relapsing-remitting multiple sclerosis (RRMS) are both kinds of autoimmune neuro-inflammatory disease driven by Th17. In EAE and RRMS mouse models, *INHBA* up-regulation was detected in serum and supernatant of spinal cord tissue of mice (62).

Th17 can be classified as pathogenic or non-pathogenic, and TGF-β1 was considered to be dispensable for generation of pathogenic Th17 (63). Notably, activin A secretion increased markedly during the pathogenic Th17 instead of non-pathogenic Th17 differentiation (62). Neutralizing activin A *in vitro* in the process of CD4^+^ T cells differentiating into Th17 led to reduced Th17 generation, lower levels of *Il23r* and *Csf2* (considered as pathogenic Th17 genes), higher levels of *Il10* and *Cd5l* (considered as non-pathogenic Th17 genes) (62). In line with this, an *in vivo* experiment discovered that T cell-derived activin A signaling was conducive to pathogenic Th17 cell-induced EAE rather than activin A signaling from microenvironment (62). Activin A might confer Th17 pathogenicity by inducing ERK activation, while TGF-β might inhibit ERK signaling (62). TGF-β1 receptor ALK5 transduction into T cells elicited milder EAE compared with activin A receptor ALK4 transduction and increased *Il10* expression could be observed, supporting that activin A and TGF-β1 favor pathogenic and non-pathogenic Th17 cell differentiation respectively (62). Despite this, role of activin A in Th17 cell differentiation remains controversial. RNA-sequencing (RNA-seq) detected down-regulation of pathogenic Th17 signatures including *Tbx21* and *Batf* in activin A treated Th17 cells, whereas non-pathogenic genes including *Ahr*, *Maf* and *Ctla4* were enriched (64). Furthermore, reconstitution of *Rag-1^-/-^* mice with activin A treated Th17 resulted in dampened EAE severity, indicating that activin A restrained pathogenic potential of Th17 cells (64). The reason for this contradiction may be the source of activin A. T cell-derived and exogenous activin A may have different functions in Th17 differentiation.

### Regulatory T Lymphocytes

Regulatory T cells (Tregs) can be divided into three main groups: thymus regulatory T cells (tTregs), peripheral regulatory T cells (pTregs) and induced regulatory T cells (iTregs) (65). iTregs include Th3 and type 1 regulatory T (Tr1) and some other types (65). TGF-β is crucial in the differentiation of naïve CD4^+^ T cells into CD4^+^ CD25^+^ Foxp3^+^ T cells (66). Studies on activin A modulating Tregs are mainly carried out around allergic airway diseases. Activin A plays an important role in pathogenicity of allergic respiratory diseases (59). In asthma patients, serum activin A levels, rather than TGF-β levels, are significantly correlated with severity of asthma. *INHBA* mRNA expression in CD4^+^ T cells of asthma patients is also increased, while *TGF-β* is not (60).

It was demonstrated that the differentiation and function of Tr1 were promoted by activin A *via* transcription factor IRF4 (54). Adoptive transfer of activin A-induced Tr1 protected against asthma (54). Semitekolou, M. et al. unraveled that activin A could induce the generation of antigen-specific CD4^+^ IL-10^+^ Tregs to suppress Th2 immune response, thus resisting allergic airway disease (61). TGF-β assisted activin A to suppress Th2 immune response, however, it functioned by inducing another Treg group, CD4^+^ CD25^+^ Foxp3^+^ Tregs (61).

Activin A also impacts on Tregs in the TME. Tumor focal radiation therapy can lead to the up-regulation of Tregs in both mice and patients (67, 68). After radiation therapy or TGF-β blockade, secretion of activin A from breast cancer cells could be promoted (56). Both TGF-β and activin A contributed to generation of Tregs in 4T1 tumors, and they acted to complement each other in terms of the final effect (56). In comparison to TSA, which expressed lower levels of *INHBA*, 4T1 expressed higher levels of *INHBA*. 4T1-bearing mice presented significant up-regulation of Tregs in response to TGF-β blockade (56). What’s more, in breast cancer patients, the expression of *INHBA* was positively correlated with tumor-infiltrating Tregs signatures (56). All of above findings indicate that activin A may induce Tregs in breast tumor microenvironment after radiotherapy. Due to the complementary action between activin A and TGF-β, dual blockade of both molecules may reverse immune suppression driven by radiation therapy (69).

## Discussion and Perspectives

**Figure 2:**
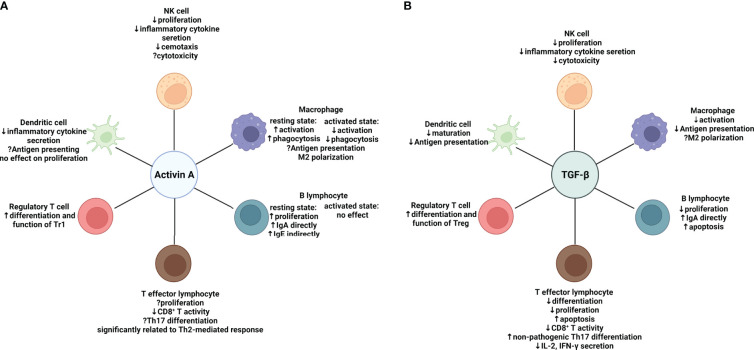
Effects of activin A and TGF-β on major components of both the innate and adaptive immune system respectively. **(A)** Effects of Activin A on the immune system: in regulation of the innate immune system, activin A suppresses proliferation, inflammatory cytokine secretion and chemotaxis of NK cells, while effects on cytotoxicity remain uncertain. The effects of activin A on macrophages depend on the activation state. Activin A promotes activation and phagocytosis of resting macrophages while suppressing that of macrophages in an activation state. Despite the elusive function in antigen-presenting, activin A seems to skew macrophages toward M2 type. Meanwhile, activin A shows inhibition of secretion of a variety of cytokines by activated DC cells. In regulation of the adaptive immune system, activin A directly induces proliferation and IgA production of resting B cells without affecting activated B cells. It can also induce IgE production indirectly by means of other immune cells *in vivo*. Activin A is significantly related to Th2-mediated response and suppressive in CD8^+^ T activity. Its role in Th17 differentiation remains controversial. In contrast to TGF-β, activin A may be inclined to induce the differentiation of IL-10-producing Tr1s. **(B)** Effects of TGF-β on the immune system. In regulation of innate immune system, TGF-β exert similar effects on NK cells to activin A It suppresses activation and antigen presentation of macrophages. More evidence is needed to prove it can induce M2-like polarization. TGF-β also inhibits maturation and antigen presentation of DCs. In regulation of adaptive immune system, TGF-β shows a wide range of immune suppressive effects. It suppresses the survival of both B and T cells. In line with activin A, it can induce IgA production by B cells and suppress CD8^+^ T activity. However, it favors differentiation of non-pathogenic Th17 and Foxp3^+^ Tregs. Suppressive effect (downward arrow); promoting effect (upward arrow); uncertain effect (question mark); NK cell, natural killer cell. Created with BioRender.com.

In this review, we focus on modulation of activin A on the major components of the immune system (**Figure 2**). Although sharing similar canonical signaling pathway, activin A functions differently from TGF-β in certain contexts, which is possibly owing to spatio-temporal distribution of the receptors, crosstalk with other pathways, and expression of related signaling molecules. Although the role of activin A in antitumor immunity remains controversial, existing evidence has proved the potential of activin A as a novel target of tumor immune therapy. On the one hand, endogenous activin A may suppress anti-tumor immunity by inducing differentiation of Tr1, deactivating innate immune cells or inhibiting cytokine secretion from CD4^+^ T cells. On the other hand, surprisingly, adoptive transfer of exogenous activin A-treated DCs or CD4^+^ T cells prevents tumor cells from growing, which provides a brand new insight on functions of TGF-β superfamily (36, 58).

However, reports on the clinical management strategy of using activin A for cancer therapy remain rare. Multiple reasons give rise to challenges for targeting activin A in tumor immune therapy, for example: presence of similarities and compensatory mechanisms with other TGF-β superfamily members, involvement in other processes of tumor development: including tumor migration, invasion or angiogenesis and tissue or cell sources for targeting. Furthermore, limited targeting specificity may lead to adverse effects and uncertainty to what extent the benefits are due to the impact of targeting activin A. As a consequence, it is critical to find approaches to identify those functions mainly performed by activin A rather than other similar ligands. Based on the immune repressive role of endogenous activin A, we expect to validate activin A as an anti-tumor factor in a wider context. Considering activin A is a pleiotropic factor, precise cell or tissue targeting design is needed to avoid affecting basic physiological functions and to maximize therapeutic efficacy as well. In addition, patient collectives suitable for receiving activin A-targeting treatment, perhaps those with poor response to immune checkpoint blockade (ICB) or high expression of INHBA in tumor microenvironment, should be clarified. Further studies will better be carried out around these aspects in order to filter out the clinical scenarios where cancer patients will receive the highest benefit with the least side effects from targeting activin A.

## Author Contributions

FL and LG conceived the topic. FL drafted the manuscript and prepared figures. Others reviewed the manuscript. All authors read and approved the final manuscript.

## Funding

This work was supported by Foundation of Shanghai Science and Technology Committee (No.22S11902100), Zhongshan Municipal Bureau of Science and Technology (No. 2020SYF08) and the Department of Science and Technology of Guangdong Province (No. 2019B090904008 and No. 2021B0909050003).

## Conflict of Interest

The authors declare that the research was conducted in the absence of any commercial or financial relationships that could be construed as a potential conflict of interest.

## Publisher’s Note

All claims expressed in this article are solely those of the authors and do not necessarily represent those of their affiliated organizations, or those of the publisher, the editors and the reviewers. Any product that may be evaluated in this article, or claim that may be made by its manufacturer, is not guaranteed or endorsed by the publisher.
